# A numerical study on nonlinear dynamics of oscillatory time-depended viscoelastic flow between infinite parallel plates: utilization of symmetric and antisymmetric Chandrasekhar functions

**DOI:** 10.1016/j.heliyon.2019.e02647

**Published:** 2019-10-23

**Authors:** Reza Roohi, Nariman Ashrafi, Sepideh Samghani, Mohammad Najafi

**Affiliations:** a Department of Mechanical Engineering, Fasa University, Fasa, Iran; b Department of Mechanical and Aerospace Engineering, Science and Research Branch, Islamic Azad University, Tehran, Iran; c Department of Mechanical Engineering, Shiraz University, Shiraz, Iran

**Keywords:** Computational mathematics, Mechanical engineering, Mechanics, Nonlinear physics, Oscillatory flow, Viscoelastic fluid, Stability analysis, Johnson–Segalman model, Chandrasekhar functions

## Abstract

The one-dimensional viscoelastic fluid flow between two infinite parallel plates with oscillatory inlet condition is examined using the Johnson–Segalman model. The symmetric and antisymmetric Chandrasekhar functions in space are utilized to represent the velocity and stress fields. The non-dimensional form of the conservation laws in addition to the constitutive equations are solved numerically based on the Galerkin projection method. Two critical Weissenberg numbers (*We*) for various Reynolds numbers (*Re*) and viscosity ratios (ε) are obtained to determine the stable range of nonlinear system behavior. Moreover, for the unsteady case, the effects of *Re*, viscosity ratio of solvent to solution as well as *We* are investigated. According to the obtained results, increasing of oscillations frequency in subcritical zone, the same as low frequency case, has almost no effect on the velocity and its gradient. Nevertheless, the normal stress amplitude of oscillations is reduced. The *Re* number determines the number of oscillations and the needed time prior to the steady condition. For lower *Re*, due to higher effect of viscosity, the initial fluctuations are intensely occurred in a short time period in contrary to the high *Re* case.

## 1 Introduction

Over the past few decades, the focus on viscoelastic fluids behavior especially in oscillatory conditions has been increased substantially due to their wide use in many industries. The significant complexities in mathematical modeling of viscoelastic fluid flow at practical scenarios is the main challenge in determination of flow characteristics.

The response of viscoelastic fluid to the oscillating force was first examined using basic models such as Maxwell model [1, 2]. In addition to the basic Maxwell model, several models based on the rheology theories are developed and implemented up to now (Bingham model [3] and Phan-Thien and Tanner (PTT) model [4]).

Moreover, Oldroyd- B model was used in [5] to study the flow of polymer dilute solvents on a flexible surface in the range of low Reynolds number. Fluid parameters including solution to solvent viscosity ratio and relaxation time was investigated using the Weissenberg number. According to their results, the flow was stable prior to reaching the maximum *We*.

One of the most familiar examples of oscillating non-Newtonian flood is blood. In the category of physiological applications, the viscoelastic nature of blood [6, 7] was investigated for low and high oscillation frequencies. The unsteady flow of blood through stenosed artery [8] was also simulated, based on an oscillatory pressure gradient as the driving force. Blood has also been treated as a non-Newtonian fluid characterized by the Oldroyd-B and Cross models [9]. According to the evaluated results, the magnitude of normal stress is reported to be much higher than shear stress and the increasing of relaxation time results in higher values of normal stress, as well.

The non-Newtonian viscoelastic fluid between parallel oscillating plates occurs in several applications namely, lubrication of mechanical devices, biomedical engineering and oil industry [10]. Fluid near the plates would oscillate harmonically with the plate in similar time period, while the amplitude of fluid motion rises slowly across the direction of the guiding surface.

Incompressible fluid flow with porosity effects are examined due to sine and cosine oscillations of infinite plates and the exact solutions were obtained for velocity field corresponding to shear stress using integral transforms techniques (Laplace and Fourier sine transformations) [11]. Based on the results, the velocity field and the shear stress are decreasing functions of oscillations amplitude. In addition, the dependence of shear stress on the affecting parameters such as relaxation and retardation time, for hydro-magnetic, viscoelastic fluid passing over an oscillating surface is investigated using Oldroyd model for free convection flow [12].

In 2017, the effect of oscillatory vibration of a thin blade on the non-Newtonian fluid flow inside a channel is numerically examined [13]. The power-law model for shear thinning and shear thicking fluid is implemented and the heat transfer enhancement due to the presence of vibrating blade is investigated.

Despite the fact that up to now, several investigations focus on the non-Newtonian fluid flow between parallel plates, the lack of a comprehensive examination regarding the flow stability analysis and parameter study is still existed. Some of the recent researches focusing on the Newtonian and non-Newtonian flow between parallel plates will be critically reviewed hereafter. Feng et al. in 2017 [14], numerically simulate the generalized Oldroyd-B fluid flow between parallel plates. The main novelty of the mentioned study is the utilization of time and space fractional calculus to expand the generality of the proposed method. However, the simulated problem is limited to moving boundary condition and the effect of fluid oscillations at the inlet is not accounted. Moreover, the investigation is merely focused on the numerical scheme proposition and the physical parameters study is the significant missing data. Keimanesh et al. [15] also conduct similar investigation on the non-Newtonian fluid between two parallel plates using the multi-step differential transform method for non-oscillatory flow condition.

The effect of parallel plates’ movement normal to the fluid flow is also examined by several researchers. Rashidi et al. in 2008 [16] tackled the problem of squeezing flow between parallel plates using the Homotopy analysis method. In another investigation, Hoshyar et al. [17] focused on unsteady incompressible Newtonian fluid flow between two parallel plates using the same method. Despite the fact these studies are dealing with the flow between parallel plates subjected to external movement effects, the non-Newtonian as well as oscillatory fluid motion is still remained as an intact expansion of the pointed out studies.

The oscillating Couette–Poiseuille flow for the Oldroyd B fluid is examined by Ma et al. in 2019 [18]. The proposed analytical method is utilized to investigate the inertial, viscous, and elastic effects on the flow behavior and stress responses. However, the range of flow stability and the bifurcation curves, which can be implemented to significantly increase the knowledge of flow behavior, is not examined in the mentioned research. Therefore, a comprehensive study of oscillatory and non-Newtonian fluid flow using Johnson–Segalman model and the flow stability analysis are still intact problems, which should be examined for specific applications.

The purpose of the present study is to investigate the steady state and transient behavior of the viscoelastic fluid with time varying inlet condition. The effect of non-linear system is considered through evaluation of time dependent governing equation with Galerkin projection method. The effect of inlet boundary condition fluctuations is investigated via introduction of oscillatory Weissenberg function. Regarding the critical Weissenberg numbers captured form the steady state solution, first the system unsteady behavior in constant Weissenberg number is obtained, then two higher and lower Weissenberg number values with respect to the critical magnitudes are chosen as the amplitude of the sinusoidal Weissenberg number for following examined cases. Moreover, the velocity and stresses plots are analyzed in various intervals of Weissenberg number. The effect of Reynolds number and polymer solute to solution viscosity ratio on velocity, normal and shear stresses are the other examined parameters.

## 2 Theory

### 2.1 The fluid flow laws

**Fig. 1:**
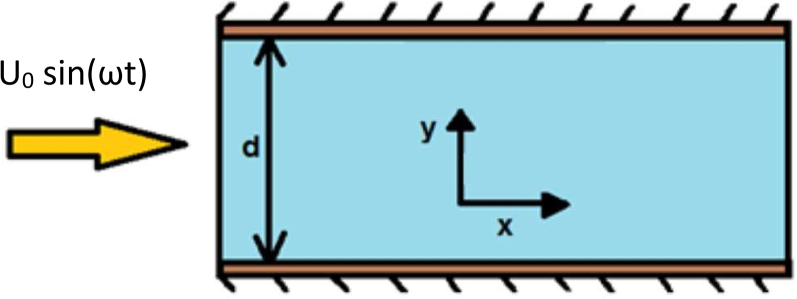
Schematic of the oscillatory flow passage through the channel.

The present study investigates the one-dimensional flow of a viscoelastic fluid between two infinite parallel plates. The plates are stationary and separated by a distance of *d*. The isothermal fluid with single relaxation time, constant viscosity and time dependent inlet velocity is examined. Moreover, the viscosity of the polymer solution (η) is determined based on the superposition of Newtonian solvent viscosity (η_1_) and the non-Newtonian polymer with viscosity of η_2_ as the solute fluid. The flow is considered as fully developed with fluctuations occur merely in one direction and no-slip condition at the walls. The schematic of the described model is illustrated in Fig. 1.

Generally, the flow behavior is governed by the conservation laws of mass and linear momentum for an incompressible fluid.

Conservation of mass:

(1) $$\frac{\partial u}{\partial x}+\frac{\partial v}{\partial y}=0$$

Conservation of momentum in x-direction

(2) $$\rho \left(\frac{\partial u}{\partial t}\right)=\frac{-\partial P}{\partial x}+\frac{\partial \tau_{xy}}{\partial y}+\eta_{2}\frac{\partial^{2}u}{\partial y^{2}}$$

where *u* and *v* are velocity components in tangential and normal directions relative to the base flow, *P* is the pressure, *t* denotes the time, *τ* is the shear stress and *η*_*2*_ is the viscosity of the solvent.

The constitutive equation is selected according to the Johnson-Segalman model as:

(3) $$\lambda \frac{DT}{Dt}+T=\eta_{1}\left[{\nabla U+\left(\nabla U\right)}^{t}\right]$$

where *λ* is a dimensionless parameter (relaxation time), *T* is stress tensor, *∇U* denotes the gradient of velocity vector and $\nabla U^{t}$ is the transpose of the *∇U*. Additionally, $\frac{DT}{Dt}$ can be calculated as:

(4) $$\frac{DT}{Dt}=\frac{\partial T}{\partial t}+U.\nabla T-\left(1-\frac{\zeta }{2}\right)\left({\left(\nabla U\right)}^{t}.T+T.\nabla U\right)+\frac{\zeta }{2}\left(\nabla U.T+T.{\left(\nabla U\right)}^{t}\right)$$

where ζ∈[0−2] is a non-dimensional fluid parameter which is a measure of non-uniform motion distribution in the stress tensor.

For the present specific case the Johnson-Segalman equation after some simplifications is converted to the following equations.

(5) $$T_{xx}=-\lambda \frac{\partial T_{xx}}{\partial t}-\lambda \left(\zeta -2\right)\frac{\partial u}{\partial y}T_{xy}$$

(6) $$T_{xy}=\eta_{1}\frac{\partial u}{\partial y}-\lambda \left\{\frac{\partial T_{xy}}{\partial t}+\frac{\zeta }{2}T_{xx}\frac{\partial u}{\partial y}+\left(\frac{\zeta }{2}-1\right)\frac{\partial u}{\partial y}T_{yy}\right\}$$

(7) $$T_{yy}=-\;\lambda \left\{\frac{\partial T_{yy}}{\partial t}+\zeta \frac{\partial u}{\partial y}T_{xy}\right\}$$

### 2.2 Non-dimensional form of the governing equations

Taking *U* to be the maximum velocity in the flow direction, the non-dimensional variables can be defined as:

(8) $$u=u^{*}\frac{d}{\lambda },\text{ ​}p=\;p^{*}\frac{\eta_{1}U}{d},\text{ ​}T=\frac{T^{*}\eta_{1}}{\lambda },\text{ ​}t=t^{*}\lambda ,\text{ ​}y=y^{*}d,\text{ ​}x=x^{*}d$$

By substitution of these transformations into Eqs. (5), (6), and (7) the following equations are formed:

(9) $$\bar{Re}\frac{\partial u}{\partial t}=\;-We\left(t\right)\;\frac{dP}{dx}+\;\frac{\partial \tau_{xy}}{\partial y}+\;\varepsilon \;\frac{\partial^{2}u}{\partial y^{2}}$$

(10) $$\frac{\partial \tau_{xx}}{\partial t}=\;-\tau_{xx}+\;\left(2-\zeta \right)\frac{\partial u}{\partial y}\tau_{xy}$$

(11) $$\frac{\partial \tau_{yy}}{\partial t}=-\tau_{yy}-\zeta \frac{\partial u}{\partial y}\tau_{xy}$$

(12) $$\frac{\partial \tau_{xy}}{\partial t}=\;-\tau_{xy}+\frac{\partial u}{\partial y}-\frac{\zeta }{2}\frac{\partial u}{\partial y}\tau_{xx}+\left(1-\frac{\zeta }{2}\right)\frac{\partial u}{\partial y}\tau_{yy}$$

The boundary condition is defined as:

(13) $$u\left(y=\mp \frac{1}{2},t\right)=0$$

Additionally, the three important similarity groups in the present problem, namely, the Reynolds number, *Re*, the Weissenberg number, *We*, the solvent to solute viscosity ratio, ε ,and the oscillatory inlet velocity are defined as:

(14) $$U=U_{{}^{\circ}\;}sin\left(\omega t\right)$$

(15) $$We\left(t\right)=\frac{U_{{}^{\circ}\;}\lambda \;}{d}sin\left(\omega t\right)$$

(16) $$\bar{Re}=\frac{d^{2}\rho }{\eta_{1}\lambda }$$

(17) $$\varepsilon =\frac{\eta_{2}}{\eta_{1}}$$

### 2.3 The base flow governing equations

The steady state condition is determined via neglecting of time derivative terms in Eqs. (9), (10), (11), and (12). The steady state stress components are obtained as follows:

(18) $$\tau_{xy}^{b}=\frac{u_{'y}}{1+2\zeta u_{'y}^{2}-\zeta^{2}u_{'y}^{2}}=\frac{u_{'y}}{1+\zeta \left(2-\zeta \right)u_{'y}^{2}}=\frac{A^{2}y^{2}}{1+\zeta \left(2-\zeta \right)A^{2}y^{2}}$$

(19) $$\tau_{xx}^{b}=\frac{\left(2-\zeta \right)u_{'y}^{2}}{1+\zeta \left(2-\zeta \right)u_{'y}^{2}}=\frac{\left(2-\zeta \right)A^{2}y^{2}}{1+\zeta \left(2-\zeta \right)A^{2}y^{2}}$$

(20) $$\tau_{yy}^{b}=\frac{-\zeta u_{'y}^{2}}{1+\zeta \left(2-\zeta \right)u_{'y}^{2}}=\frac{-\zeta A^{2}y^{2}}{1+\zeta \left(2-\zeta \right)A^{2}y^{2}}$$

where the *A* function is defined as:

(21) $$A=\frac{We\left(t\right)}{\varepsilon }\frac{dP}{dx}$$

Eqs. (5), (6), and (7) can be rewritten by the definition of normal stresses difference (*N*) and a combination of normal stresses (*Z*) as:

(22) $$\begin{array}{ccc} N=\tau_{xx}-\tau_{yy} & , & Z=\frac{\zeta }{2}\tau_{xx}+\left(1-\frac{\zeta }{2}\right)\tau_{yy} \end{array}$$

Moreover, to investigate the created disturbances in the flow, the following modifications are applied:

(23) $$\begin{array}{ccc} u=u^{b}+u^{'}, & \tau_{ij}=\tau_{ij}^{b}+\tau_{ij}^{'}, & p=p^{b}+p^{'}, \end{array}$$

For simplicity, the first term on the right-hand side of Eq. (9) is redefined as:

(24) $$B\left(t\right)=-We\left(t\right)\frac{dP}{dx}$$

Considering the relation between *A* and *B* parameters and the implementation of Eq. (23) modifications into Eqs. (9), (10), (11), and (12), the following governing equations are obtained:

(25) $$\bar{Re}u_{'t}=S_{'y}+S_{'y}^{b}+\varepsilon u_{'yy}$$

(26) $$N_{'t}=-N+2\left(A\left(t\right)yS+u_{'y}S^{b}+u_{'y}S\right)$$

(27) $$S_{'t}=-S+u_{'y}+\alpha \left(A\left(t\right)yN+u_{'y}N^{b}+u_{'y}N\right)$$

All of the unknown parameters in Eqs. (25), (26), and (27) are defined as:

(28) $$N^{b}=\frac{2{u_{'y}^{b}}^{2}}{1+\zeta \left(2-\zeta \right){u_{'y}^{b}}^{2}}$$

(29) $$S^{b}=\frac{u_{'y}^{b}}{1+\zeta \left(2-\zeta \right){u_{'y}^{b}}^{2}}$$

(30) $$u_{'y}^{b}=Ay$$

(31) $$A=\frac{We}{\varepsilon }\frac{dP}{dx}$$

(32) $$\alpha =\;\zeta \left(\frac{\zeta }{2}-1\right)$$

### 2.4 Solution method

The solution of Eqs. (25), (26), and (27) is determined using the Galerkin projection method. To do so, *U(y,t)*, *S(y,t)* and *N(y,t)* are introduced by Chandrasekhar Function which satisfies the no-slip boundary condition.

Therefore, the general series representation of the velocity and stress differences is given by Eqs. (33), (34), and (35) as:

(33) $$u\left(y,t\right)=\sum_{i=1}^{M}U_{i}\left(t\right)\phi_{i}\left(y\right)$$

(34) $$N\left(y,t\right)=\sum_{i=1}^{M}N_{i}\left(t\right)\phi_{i}\left(y\right)$$

(35) $$S\left(y,t\right)=\sum_{i=1}^{M}S_{i}\left(t\right)\phi_{i}^{'}\left(y\right)$$

where $\phi_{i}\left(y\right)$ is the even and odd Chandrasekhar functions, for i even and odd, respectively [12], $\phi_{i}^{'}\left(y\right)$ are defined as $\phi_{i}^{'}\left(y\right)=\left(1/\alpha_{i}\right)\left(d\phi_{i}/dy\right)$, where $\alpha_{i}$ are constants and *M* is the number of modes. The suitable level of truncation that is imposed hereafter in the calculations is *M* = 2 which leads to a six-dimensional model.

The first step in Galerkin projection method is the substitution of Eqs. (33), (34), and (35) into Eqs. (25), (26), and (27). Then each equation is multiplied by the suitable mode and the integration is performed in the range related to the problem geometry (et. y∈[−12−12]). Hence, a set of ordinary, coupled and non-linear differential equations are derived with time dependent coefficients.

The projection method leads to explicit expressions for time derivatives (et. ${\text{u}}_{\text{k}}$ and ${\text{N}}_{\text{k}}$ where k∈[1−M]):

(36) $$\bar{Re}\frac{\partial U_{1}}{\partial t}=S_{1}\alpha_{1}D_{1}+S_{2}\alpha_{2}D_{2}+\varepsilon U_{1}\alpha_{1}^{2}D_{1}+\varepsilon U_{2}\alpha_{2}^{2}D_{2}+E_{1}$$

(37) $$\bar{Re}\frac{\partial U_{2}}{\partial t}=S_{1}\alpha_{1}D_{3}+S_{2}\alpha_{2}D_{4}+\varepsilon U_{1}\alpha_{1}^{2}D_{3}+\varepsilon U_{2}\alpha_{2}^{2}D_{4}+E_{2}$$

(38) $$\frac{\partial N_{1}}{\partial t}=-N_{1}+2A\left(t\right)S_{1}D_{5}+2A\left(t\right)S_{2}D_{6}+2\alpha_{1}U_{1}D_{10}+2\alpha_{2}U_{2}D_{11}+2\alpha_{1}U_{1}S_{1}D_{7}+2\alpha_{1}U_{1}S_{2}D_{8}+2\alpha_{2}U_{2}S_{1}D_{8}+2\alpha_{2}U_{2}S_{2}D_{9}$$

(39) $$\frac{\partial N_{2}}{\partial t}=-N_{2}+2A\left(t\right)S_{1}D_{12}+2A\left(t\right)S_{2}D_{13}+2\alpha_{1}U_{1}D_{14}+2\alpha_{2}U_{2}D_{15}+2\alpha_{1}U_{1}S_{1}D_{16}+2\alpha_{1}U_{1}S_{2}D_{17}+2\alpha_{2}U_{2}S_{1}D_{17}+2\alpha_{2}U_{2}S_{2}D_{18}$$

(40) $$\frac{\partial S_{1}}{\partial t}=-S_{1}+\alpha_{1}U_{1}\left(1+\alpha E_{3}\right)+\alpha \alpha_{2}U_{2}E_{4}+\alpha A\left(t\right)N_{2}D_{19}+\alpha \alpha_{1}U_{1}N_{1}D_{20}+\alpha \alpha_{2}U_{2}N_{2}D_{21}$$

(41) $$\frac{\partial S_{2}}{\partial t}=-S_{2}+\alpha_{2}U_{2}\left(1+\alpha E_{5}\right)+\alpha A\left(t\right)N_{1}D_{22}+\alpha \alpha_{1}U_{1}E_{6}+\alpha \alpha_{1}U_{1}N_{2}D_{23}+\alpha \alpha_{2}U_{2}N_{1}D_{24}$$

The coefficients in Eqs. (36), (37), (38), (39), (40), and (41) are defined as:

(42) D1=∫−1212∅1pp∅1dyD2=∫−1212∅2pp∅1dyD3=∫−1212∅1pp∅2dyD4=∫−1212∅2pp∅2dyE1=∫−1212S'yb∅1dyE2=∫−1212S'yb∅2dyD5=∫−1212∅1p∅1dyD6=∫−1212y∅2p∅1dyD7=∫−1212∅1p∅1p∅1dyD8=∫−1212∅1p∅2p∅1dyD9=∫−1212∅2p∅2p∅1dyD10=∫−1212∅1p∅1SbdyD11=∫−1212∅2p∅1SbdyD12=∫−1212y∅1p∅2dyD13=∫−1212y∅2p∅2dyD14=∫−1212∅1p∅2SbdyD16=∫−1212∅1p∅1p∅2dyD15=∫−1212∅2p∅2SbdyD17=∫−1212∅1p∅2p∅2dyD18=∫−1212∅2p∅2p∅2dyD19=y(∅2∅1p)(∅1p∅1p)D20=(∅1p∅1∅1p)(∅1p∅1p)D21=(∅2p∅2∅1p)(∅1p∅1p)D22=y(∅1∅2p)(∅2p∅2p)D23=(∅1p∅2∅2p)(∅2p∅2p)D24=(∅2p∅1∅2p)(∅2p∅2p)E3=Nb(∅1p∅1p)(∅1p∅1p)E4=Nb(∅2p∅1p)(∅1p∅1p)E5=Nb(∅2p∅2p)(∅2p∅2p)E6=Nb(∅1p∅2p)(∅2p∅2p)

where $\emptyset_{i}^{pp}$ denotes second derivative of $\emptyset_{i}$. The () operator in Eq. (42) is defined as:

(43) (ab)=∫−1212ab ​ⅆy

Briefly, the nonlinear governing equations are transformed into a set of simultaneous ordinary differential equations (ODE) using the Galerkin method (Eqs. (36), (37), (38), (39), (40), and (41)). The coefficients of the ODE set can be obtained using the integration of the Chandrasekhar function modes. The numerical simulation of the Mathematica software is performed with the aid of a computer system with following characteristics: Intel Core i7 CPU@2.8GHz, 16 GB of DDR4 RAM. Each simulation is converged in about 3 min to the final results.

## 3 Results & discussion

### 3.1 The bifurcation graph

In the present section the numerical solution of the governing equations (Eqs. (36), (37), (38), (39), (40), and (41)) is presented for various *ξ* and *We* numbers. The variations of velocity, normal and shear stresses (namely *U*_*1*_, *U*_*2*_, *N*_*1*_, *N*_*2*_, *S*_*1*_ and *S*_*2*_) are calculated for We∈[0−10] and δ∈[0.2−1.0] for limiting *Re* values of 0.2 and 10. It should be added that Δp and *ε* parameters are set to -1 and 0.04, respectively.

**Fig. 2:**
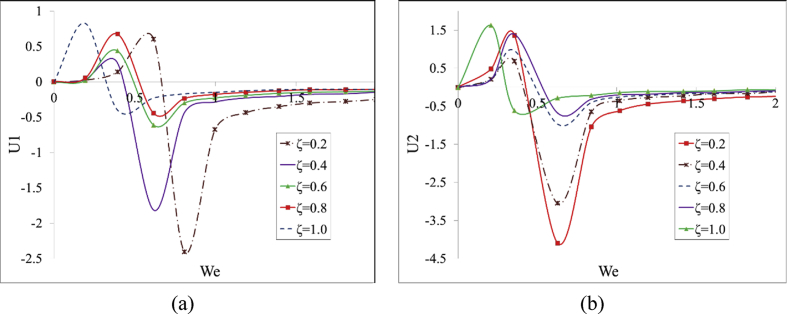
The variations of a) *U*_*1*_, and b) *U*_*2*_ as a function of *We* for ε=0.04, ​Re=0.2 ​ and Δp=−1.

According to Fig. 2 (the bifurcation graph), the *U*_*1*_ variable has two sequential maximum and minimum values for relatively low *We* numbers which is due to the disturbance terms in Eq. (23) (*i.e.* u', p' and ${\text{τ}}_{\text{ij}}^{\text{'}}$). As the value of these peaks is reduced the fluid resistance to the imposed forces and initial disturbances is decreased. Based on Fig. 2, three main zones can be defined as subcritical ($We<We_{C1}$), critical ($We_{C1}<We<We_{C2}$) and supercritical ($We>We_{C2})$.

First and second critical *We* numbers are located in the range of [0.2−0.6] and [0.4−0.8], respectively. Unstable base flow with nonlinear velocity profile is observed between these two critical *We* numbers.

### 3.2 Validation

**Fig. 3:**
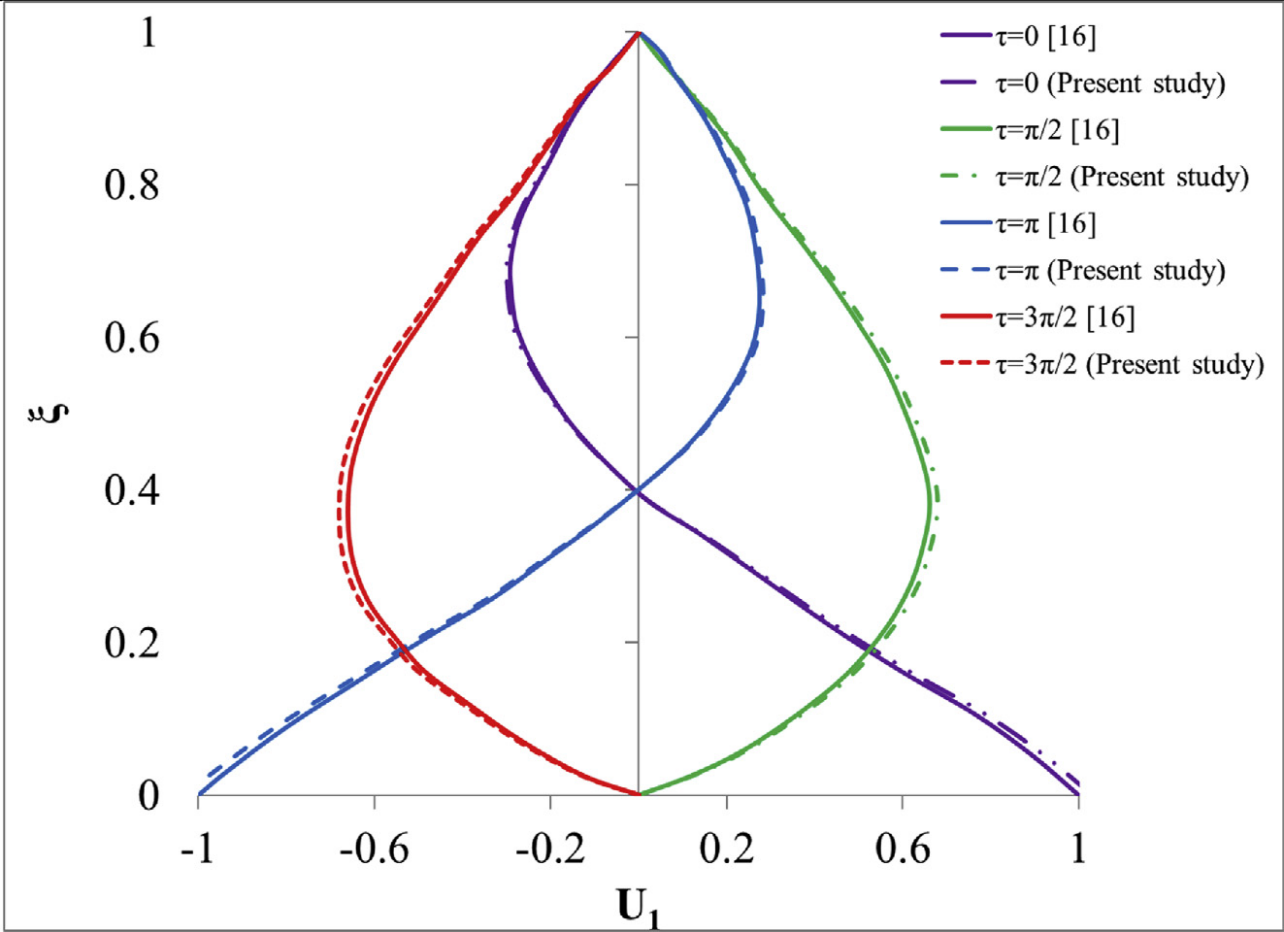
Velocity profile for oscillating Couette–Poiseuille flow of upper-convected Maxwell fluid at various time instances.

It should be noted that, the oscillatory problem of current study using the Johnson–Segalman model is not tackled in the available literature yet. Thus, the validation is performed by the analytical data of oscillating Couette–Poiseuille flow of upper-convected Maxwell fluid of Ma et al. [18], which has approximately similar problem definition in comparison to the present study. The velocity profile for Re = 10 at four different time instances during one time period of oscillations are calculated and illustrated in Fig. 3.

It should be noted that the ξ and π parameters are dimensionless depth and time variables, respectively. According to the results, it can be concluded that the velocity profile of the current study is in close agreement with the analytical data of [18]. The difference between the numerical results of the current study with the analytical data is below 4% at all of the time instances through the channel depth.

### 3.3 Transient simulation results

To examine the nonlinear behavior of the system in the transient period, the time dependent outputs of the governing equation from the initial condition is studied. The effect of boundary fluctuations is considered by dedication of a sinusoidal function to the *We* (Eq. 15). To do so, two *We* numbers in the stable sub/supercritical zones are selected based on the steady solution (et. 0.15 and 15) and the variations of velocity and stress components are examined for both constant and oscillatory *We* numbers. Moreover, the *Re* number is assumed to be 0.2 while the case of *Re* = 10 is also investigated to determine the effect of *Re* number on the fluid flow characteristics.

**Fig. 4:**
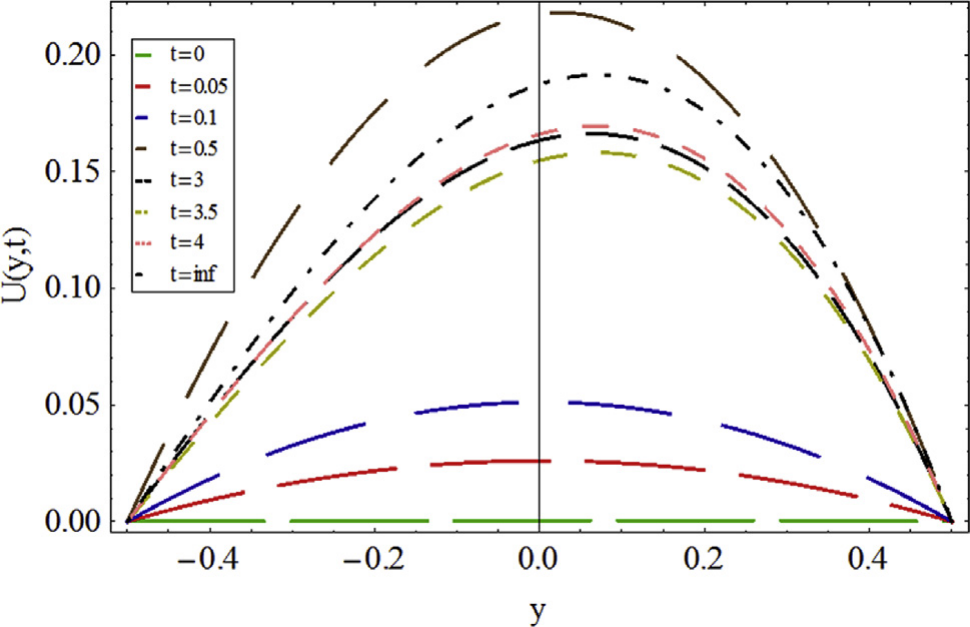
The transient behavior of flow in the channel in subcritical zone for Re=0.2, We=0.15, Δp=−1, ε=0.04 and ζ=0.2.

The velocity profile variations from the initial condition to the steady configuration is presented in Fig. 4. The velocity profiles approximately resemble the Newtonian velocity profile, although a slight declination to the right is observed in the profiles.

Considering the final configuration of the velocity profiles, it can be stated that from t = 0 to t = 3 the velocity magnitude is overgrowing, while it is decreased slightly to t = 4 when the final profile is almost shaped.

**Fig. 5:**
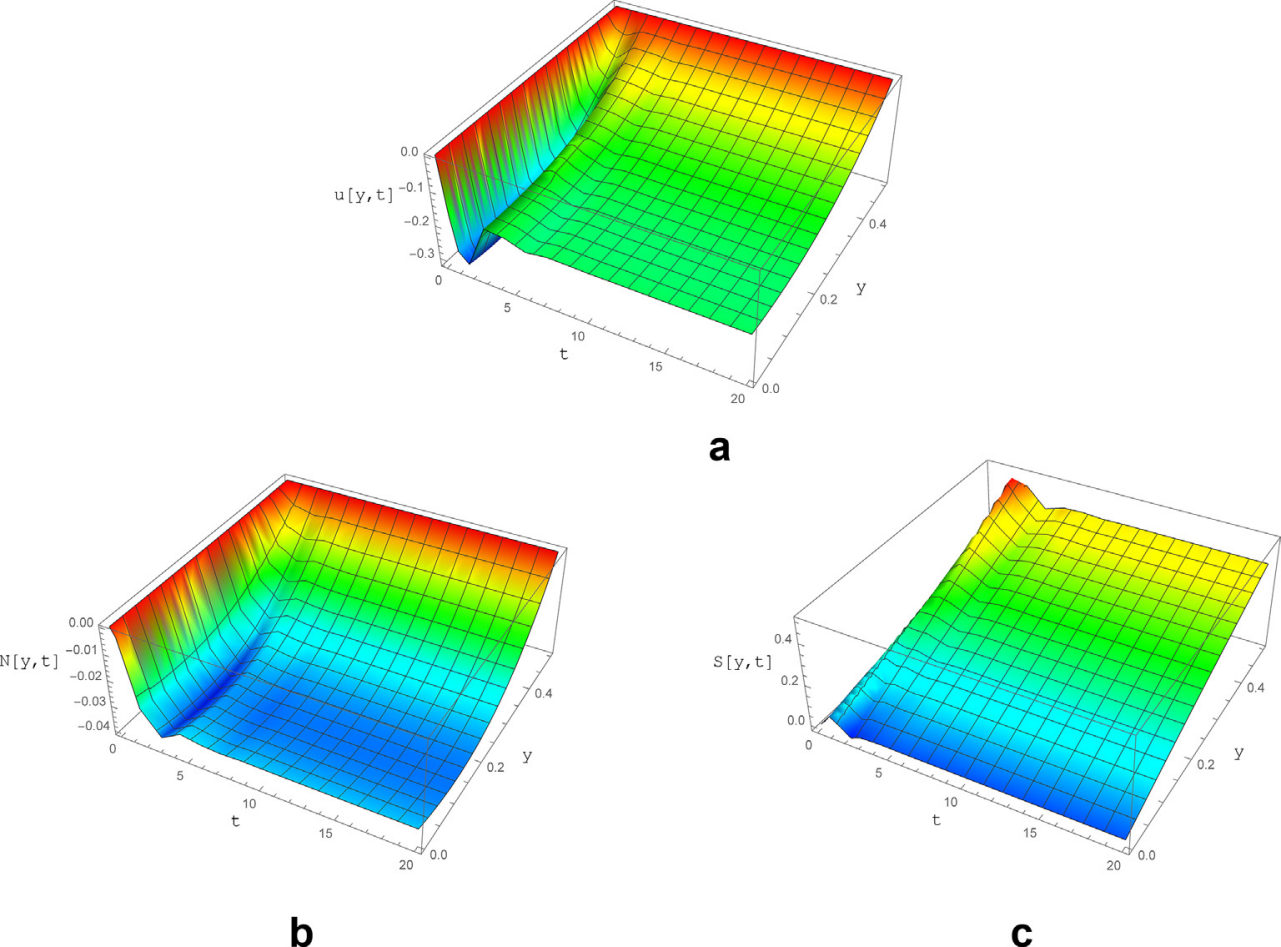
The variations of a) velocity, b) normal and c) shear stress as a function of y and t for subcritical *We* with parameters of ε=0.04,  ​Re=0.2, ∇p=−1, We = 0.15 and, ζ = 0.2.

For better visualization, the y-t-u diagram is presented in Fig. 5. Considering the results, the fluid has a tendency to fluctuate immediately after the initial condition. However, the oscillations are damped to zero after limited waves near t = 4. In addition, it should be noted that the velocity is set to zero at the walls due to the no-slip condition.

The normal stress is equal to zero at channel walls and has an increasing trend marching toward the channel midsection, where the maximum is occurred at y = 0.12. Moreover, the early fluctuations as well as the existence of steady state after t = 4.5 is noticeable.

The shear stress as it is expected, has its maximum near the wall (y = 0.5), which is intensified until t = 2.0. Subsequently, the shear stress maximum is reduced slightly toward its stable value (t > 4.0), which is similar to the Newtonian flow.

**Fig. 6:**
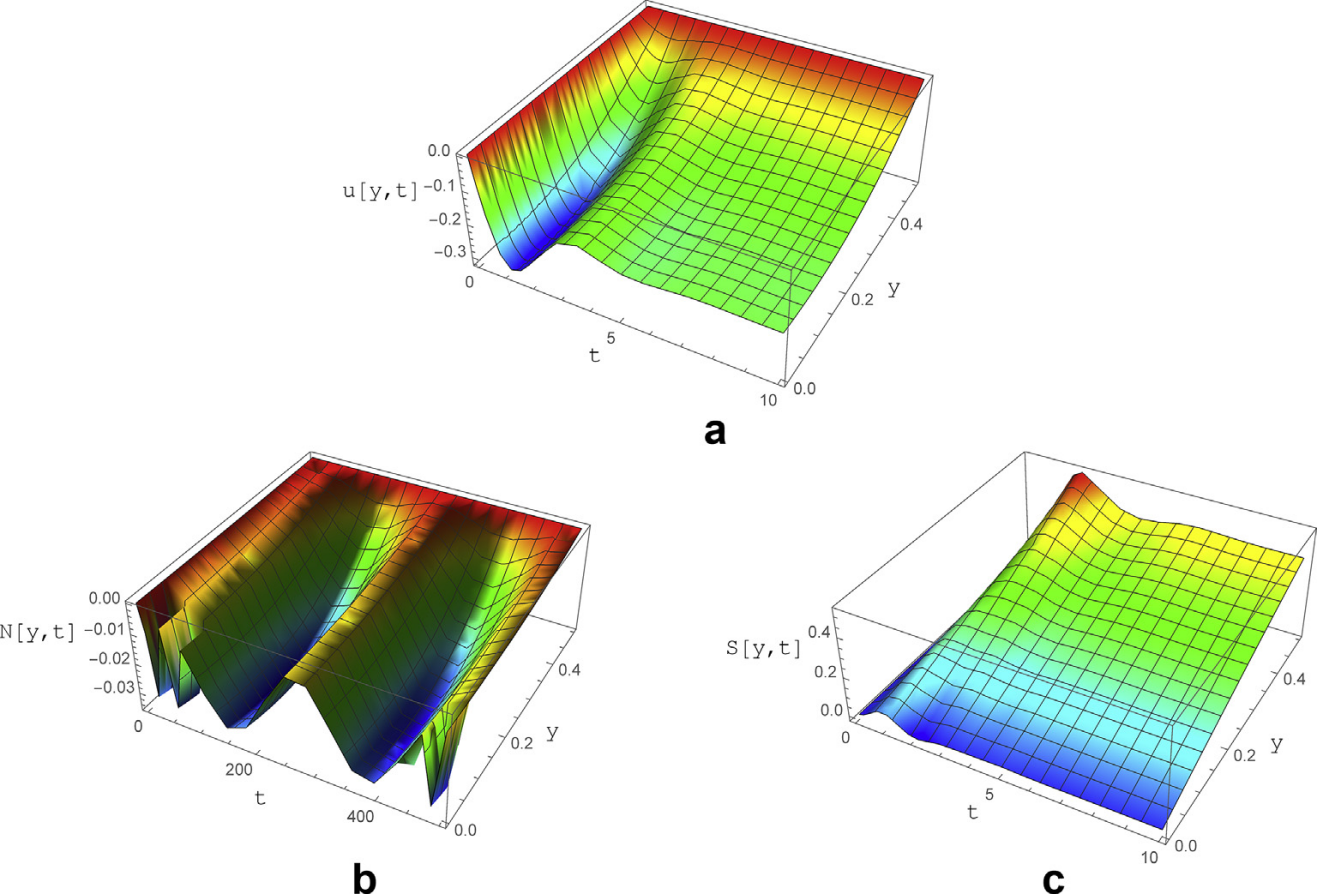
The variations of a) velocity, b) normal and c) shear stress as a function of y and t for subcritical *We* with parameters of ε=0.04, Re=0.2, ∇p=−1, We=0.15*Sin(0.5t) and ζ = 0.2.

The effect of temporal oscillations of the inlet velocity magnitude is illustrated in Fig. 6. The oscillatory behavior of the inlet boundary is simulated for low and high frequencies (*i.e.* sin (0.5t) and sin (20t)). It can be observed that the low frequency variations of the inlet velocity magnitude have the same effect as the non-fluctuating boundary condition. The same trend can be seen for the shear stress, which leads to the conclusion that the Weissenberg fluctuations especially at low frequencies has negligible effect on both velocity and its derivatives (Fig. 6).

In contrary to the velocity and shear stress, the normal stress is completely affected by the low frequency oscillations of *We*. The normal stress fluctuations have variable time periods from 50 to 200. The effect of inlet sinusoidal *We* on the damping and exciting terms in the *N* equation (Eqs. (38) and (39)) is the reason for such a behavior.

**Fig. 7:**
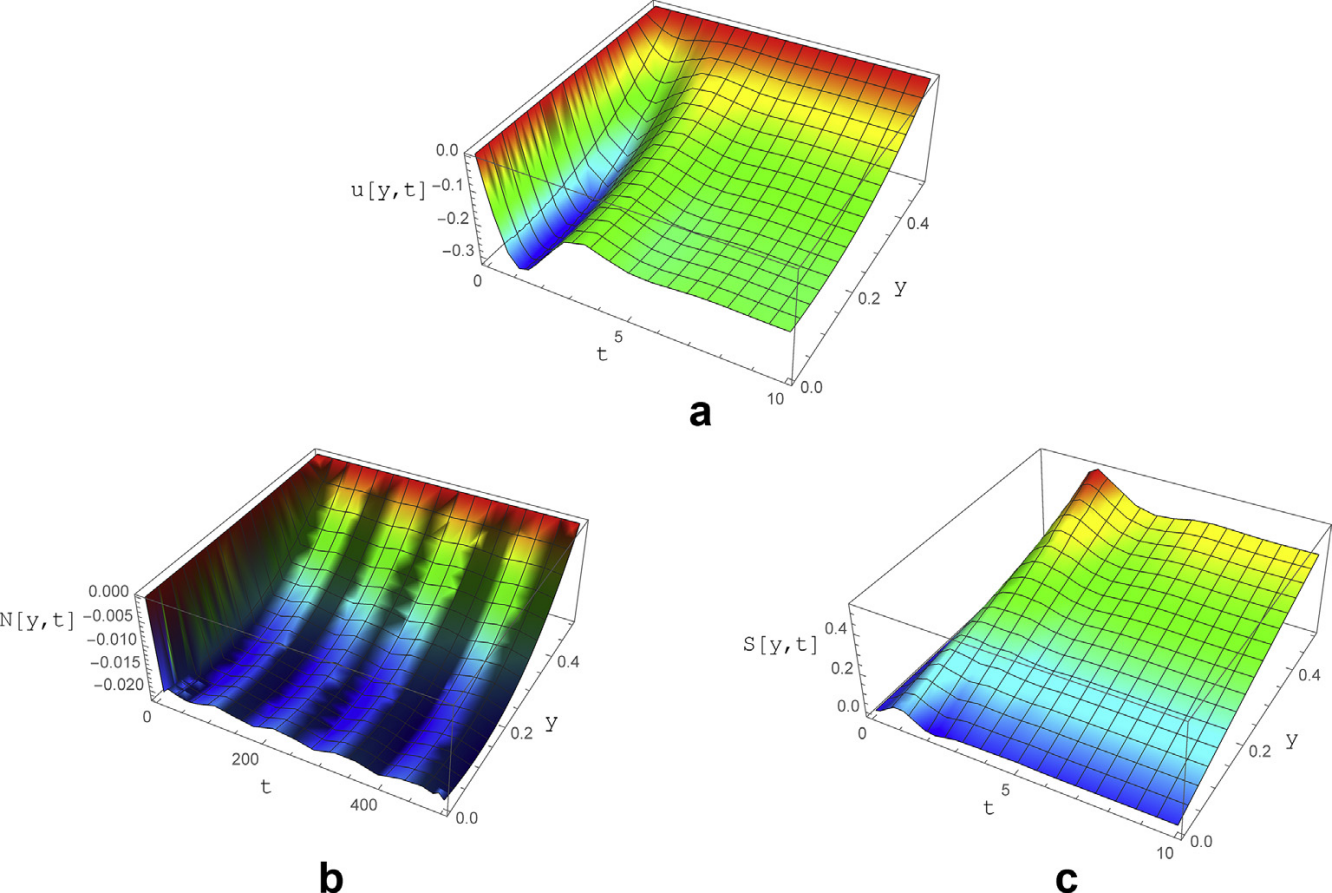
The variations of a) velocity, b) normal and c) shear stress as a function of y and t for subcritical zone with parameters of ε=0.04, Re=0.2, ∇p=−1, We=0.15*Sin(20t) and ζ = 0.2.

The effect of increasing of the oscillation frequency is presented in Fig. 7. The amplitude of *We* is chosen to be 0.15 and its frequency is set to be 20/2π. Velocity and shear stress behaviors are similar to the previous case with inlet velocity frequency of 0.5/2π (Fig. 6(a) and (c)). However, the amplitude of normal stress fluctuations significantly reduced (to about 0.005) with the period of 100. After t = 30 the oscillations turn into stable periodic waves with a peak value (0.022) slightly lower than the previous case (0.032) with lower oscillation frequency (Fig. 6).

**Fig. 8:**
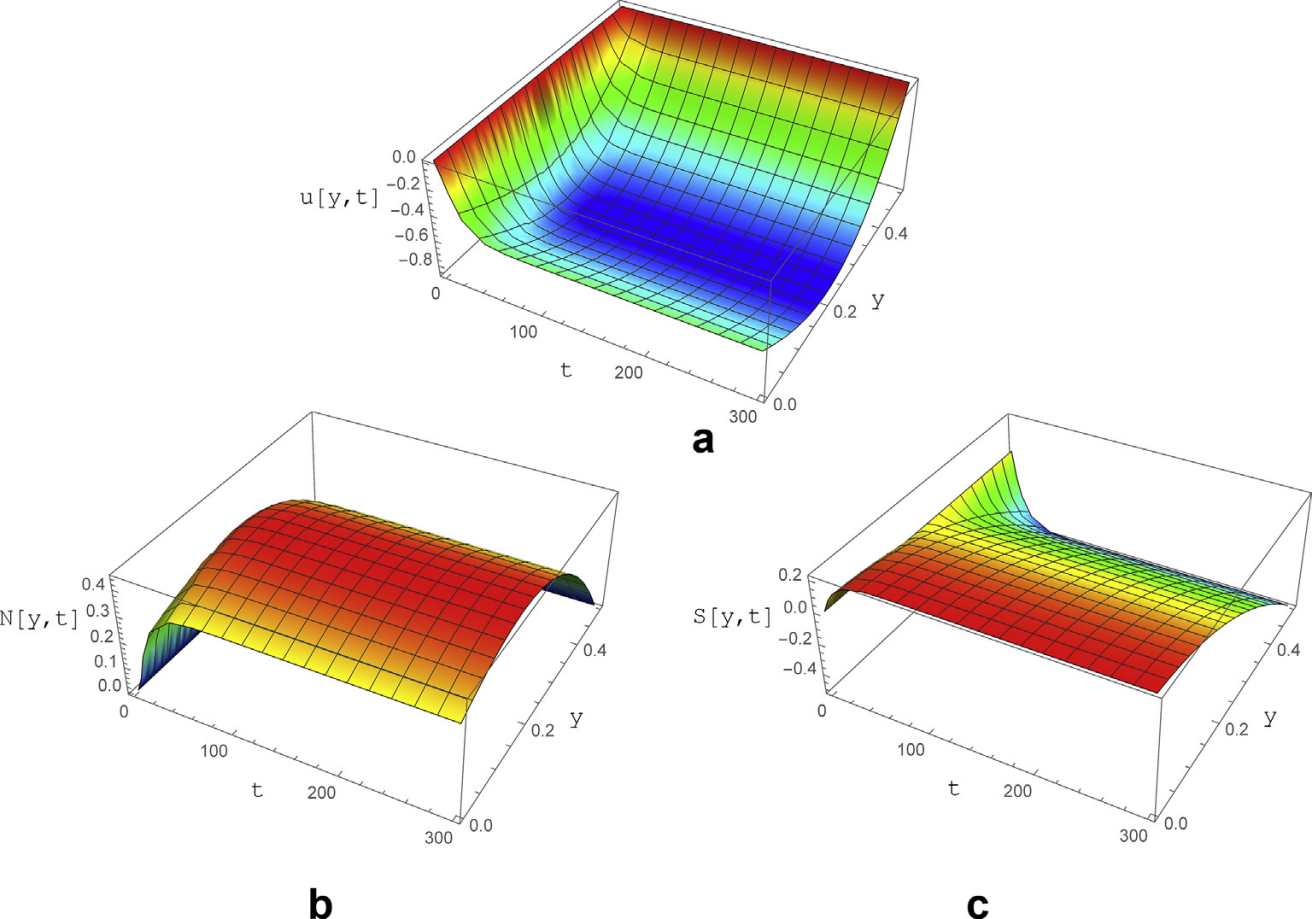
The variations of a) velocity, b) normal and c) shear stress as a function of y and t for subcritical zone with parameters of ε=0.04, Re=0.2, ∇p=−1, *We* = 15 and ζ = 0.2.

In the current section the characteristics of the viscoelastic flow for *We* variations (constant and low frequency oscillations) is examined in the supercritical range. With the operational conditions of *We* = 15, *ε* = 0.04 and *Re* = 0.2, it is depicted in Fig. 8, that the velocity has a non-oscillatory behavior and will be uniform after about t = 30, similar to the normal and shear stress diagrams. Comparison to related figures for subcritical condition (Fig. 5), one can deduce that the location of maximum velocity relocated from channel center to near walls and the required time to stabilization of the flow is substantially increased.

The normal stress increases till t = 30 after which a nearly uniform trend is observed with its highest value occurred at y = 0.2. It is worth noting that in contrary to the subcritical case, the normal stress is positive for *We* > *We*_*C1*_.

**Fig. 9:**
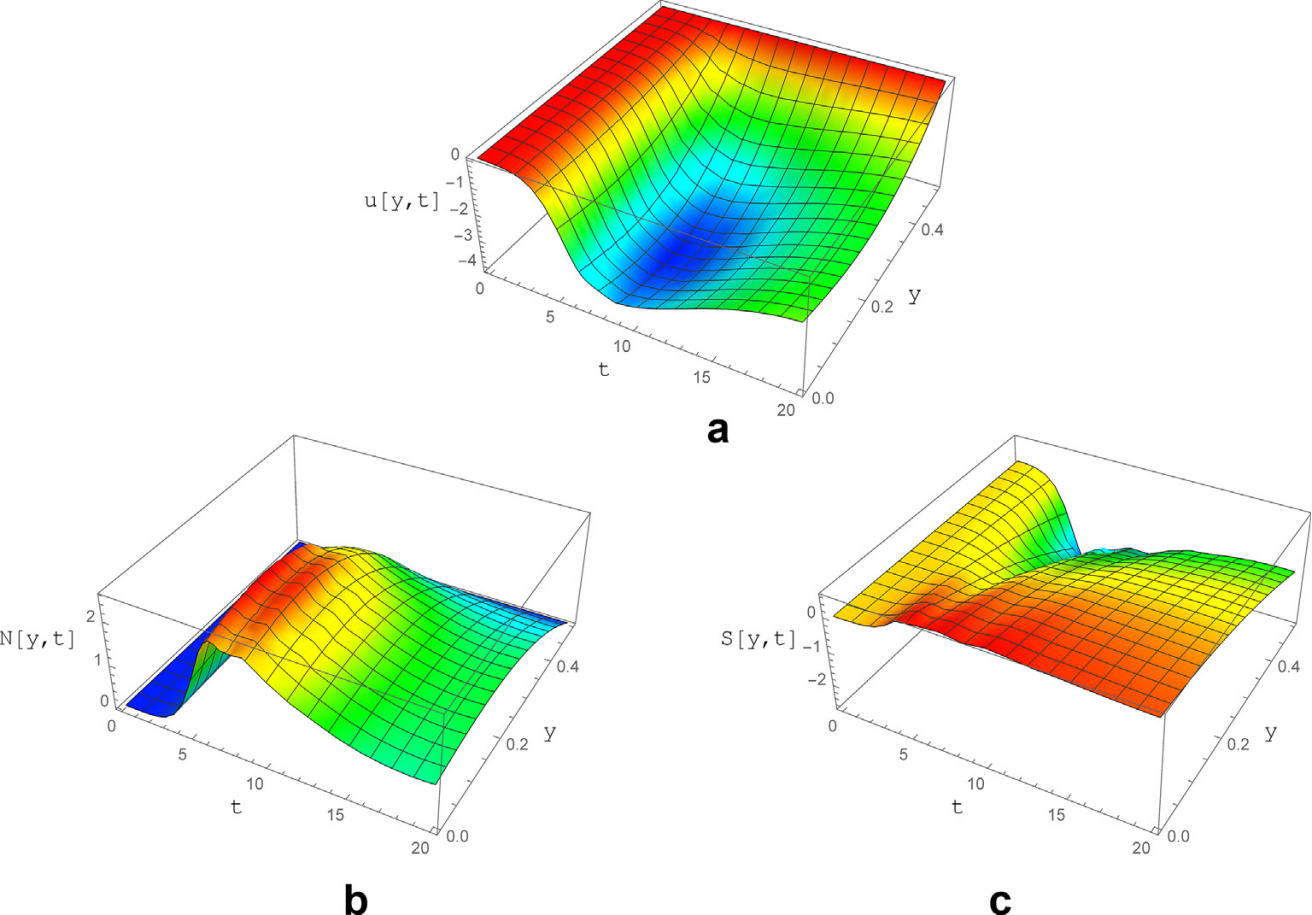
The variations of a) velocity, b) normal and c) shear stress as a function of y and t for subcritical zone with parameters of ε=0.04, Re=0.2, ∇p=−1, We=15*Sin(0.1t) and, ζ = 0.2.

Finally, the effect of boundary fluctuations on the viscoelastic flow characteristics in supercritical zone is investigated. According to Fig. 9(a) the velocity magnitude initially increased and after t = 10 a slight reduction to its steady profile at t = 20 occurred with velocity maximum located at y = 0.2.

The normal stress variable is depicted in Fig. 9(b). Except of two instabilities between t = 5 and t = 10, the flow is almost stable with zero value of normal stress at the channel walls and maximum at y = 0.2. Regarding the shear stress in the supercritical range of *We*, after some pulsation for t = 5 to 10, its magnitude reaches the final value (Fig. 9-c).

For the supercritical case especially at high oscillations frequencies, the flow field becomes unstable. The reason of such behavior is due to the relative increase of the *We* in comparison to *ε* parameter, which in turn enhances the magnitude of non-linear terms and leads to instability of flow field.

### 3.4 The effect of Re on the transient behavior of flow parameters

**Fig. 10:**
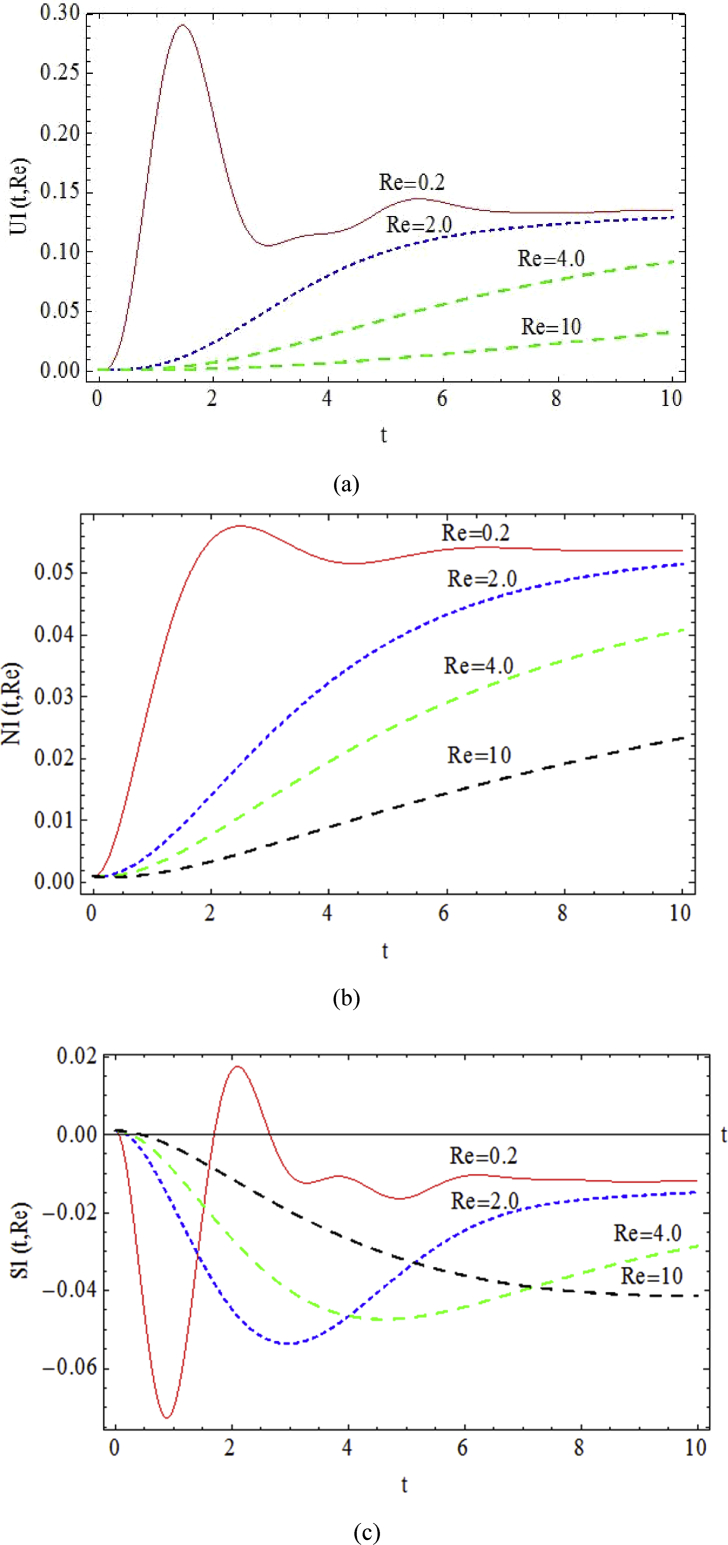
Transient behavior of a) *U1*, b) *N1* and c) *S1*, for various *Re* with *We* = 0.15, *Δp* = -1, *ζ* = 0.2 and *ε* = 0.04.

The transient response of the flow parameters (*U*_*1*_, *N*_*1*_ and *S*_*1*_) for *We* = 0.15, *Δp* = -1, *ζ* = 0.2 and *ε* = 0.04 to the variations of *Re* number is presented in Fig. 10. For small *Re* the convergence to steady condition due to viscosity effect is uniform and rapid with several high amplitude oscillations. In the case with Re = 0.2 after three consecutive vibrations at t = 8 the variables become steady, however the mentioned steady state condition doesn't occur before t = 10 for Re = 4. The numerical data show the same trend for stress components. Therefore, the *Re* number determines the number of oscillations as well as convergence rate prior to the stable condition.

### 3.5 The effect of *ε* on the transient behavior of flow parameters

The effect of *ε* (the viscosity ratio of solvent to soluble) on the velocity, shear and normal stress is examined in the present section.

**Fig. 11:**
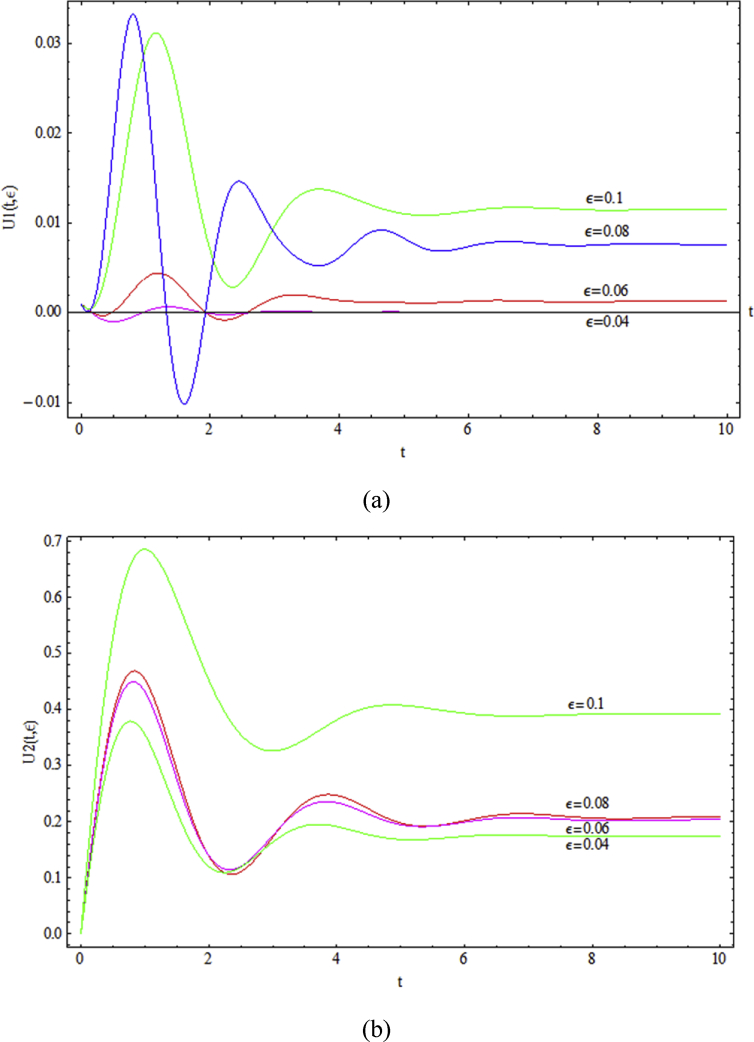
Transient behavior of a) *U*_*1*_ and b) *U*_*2*_, for various *ε* with *We* = 0.15, *Δp* = -1, *ζ* = 0.2 and *Re* = 0.2.

According to *U*_*1*_ and *U*_*2*_ curves which are obtained for *We* = 0.15 and *Re* = 0.2, by increasing the *ε* parameter from 0.04 to 0.1 the stable velocity magnitude as well as initial fluctuations and the time needed for the system to reach steady state is increased (Fig. 11).

Besides, increasing of *ε* by the assumption of constant viscosity for the Newtonian solute (water), means a reduction in the viscosity of the Non-Newtonian soluble. Hence, the pointed out growths are meaningful as a results of reduction of solution viscosity and its lower capability in damping of the flow fluctuations.

**Fig. 12:**
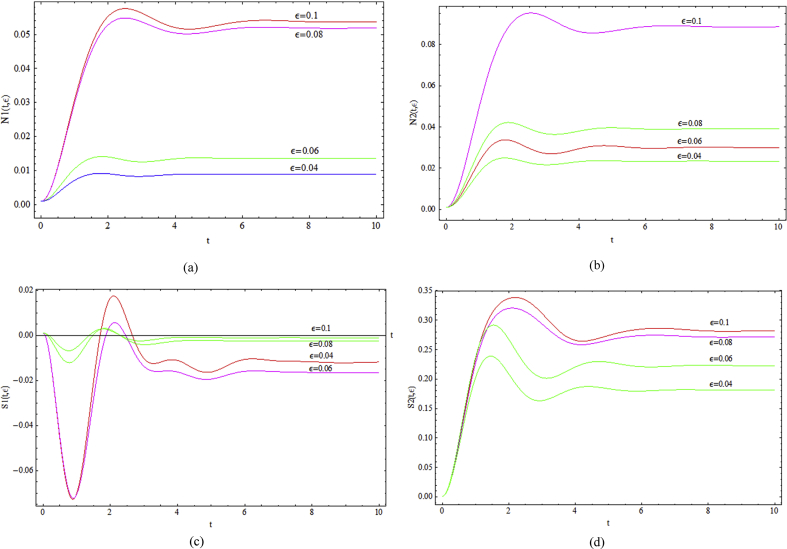
Transient behavior of a) *N*_*1*_, b) *N*_*2*_, c) *S*_*1*_ and d) *S*_*2*_, for various *ε* with *We* = 0.15, *Δp* = -1, *ζ* = 0.2 and *Re* = 0.2.

Increasing of *ε* results in generation of higher normal stresses. Moreover, the maximum and minimum peaks at the initial time are intensified. The same observation can be detected for the shear stress (Fig. 12).

## 4 Conclusion

1) The examination of the governing equations of the viscoelastic flow with Johnson-Segalman model denotes that the flow behavior between two critical *We* numbers becomes unstable and the nonlinear velocity profile emerges.
2) The inspection of the *Re* number effect implies that by enhancement of *Re*, the flow becomes stable for all of the simulated *We* and the early velocity and stress peaks diminish.
3) In the subcritical zone, the velocity profile for non-oscillatory viscoelastic fluid is similar to the Newtonian fluid. However, the velocity and stress profiles overgrowing the stable profile at some early instances and damp to the final condition afterwards.
4) Increasing of oscillations frequency in subcritical zone, the same as low frequency case, has almost no effect on the velocity and its gradient. Nevertheless, the normal stress amplitude of oscillations is reduced.
5) For non-oscillatory, supercritical case in contrary to the subcritical situation in which the velocity maximum occurs at the midline, its location shifted toward the walls and the stabilization period increased.
6) The *Re* number determines the number of oscillations and the needed time prior to the steady condition. For lower *Re*, due to higher effect of viscosity the initial fluctuations are intense and occurred in a short time period in contrary to the high *Re* case.

## Declarations

### Author contribution statement

Reza Roohi, Nariman Ashrafi, Sepideh Samghani, Mohammad Najafi: Conceived and designed the analysis; Analyzed and interpreted the data; Contributed analysis tools or data; Wrote the paper.

### Funding statement

This research did not receive any specific grant from funding agencies in the public, commercial, or not-for-profit sectors.

### Competing interest statement

The authors declare no conflict of interest.

### Additional information

No additional information is available for this paper.

## References

[1] Thurston G.B. (1959). Theory of oscillation of a viscoelastic medium between parallel planes. J. Appl. Phys..

[2] Del Río J.A., Lopez de Haro M., Whitaker S. (2001). Enhancement in the dynamic response of a viscoelastic fluid flowing in a tube. Phys. Rev. E..

[3] Thurston G.B. (1994). Non-Newtonian viscosity of human blood: flow-induced changes in microstructure. Biorheology.

[4] Piau M. (2002). Easier flow of viscoplastic materials with ultrasonic longitudinal wall motion. J. Non-Newtonian Fluid Mech..

[5] Cruz F.T., Pinho P.J., Oliveira c. (2005). Analytical solutions for fully developed laminar flow of some viscoelastic liquids with a Newtonian solvent contribution D.O.A. J. Non-Newtonian Fluid Mech..

[6] Roohi R., Emdad H., Jafarpur K. (2016). Performing effective drug delivery and hyperthermia based on biological and treatment parameters: a comprehensive Eulerian-Lagrangian Approach. J. Comput. Theor. Nanosci..

[7] Roohi R., Emdad H., Jafarpur K. (2019). “A comprehensive study and optimization of magnetic nanoparticle drug delivery to cancerous tissues via external magnetic field‏”. J. Test. Eval..

[8] Chokshi P., Kumaran V. (2007). Stability of the Flow of a Viscoelastic Fluid Past a Deformable Surface in the Low Reynolds Number Limit.

[9] Ghigo A.R., Lagrée P.Y., Fullana J.M. (2018). A time-dependent non-Newtonian extension of a 1D blood flow model. J. Non-Newtonian Fluid Mech..

[10] Roohi R., Heydari M.H., Bavi O., Emdad H. (2019). Chebyshev polynomials for generalized Couette flow of fractional Jeffrey nanofluid subjected to several thermochemical effects. Eng. Comput..

[11] Shahid N., Rana M., Siddique I. (2012). Exact solution for motion of an Oldroyd-B fluid over an infinite flat plate that applies an oscillating shear stress to the fluid. Bound. Value Probl..

[12] Kashif A. (2016). Porous effects on second grade fluid in oscillating plate. J. Appl. Environ. Biol. Sci..

[13] Izadpanah E., Babaie M., Sadeghi H., Talebi S. (2017). Effect of rotating and oscillating blade on the heat transfer enhancement of non-Newtonian fluid flow in a channel. Appl. Therm. Eng..

[14] Feng L., Liu F., Turner I., Zhuang P. (2017). Numerical methods and analysis for simulating the flow of a generalized Oldroyd-B fluid between two infinite parallel rigid plates. Int. J. Heat Mass Transf..

[15] Keimanesh M., Rashidi M.M., Chamkha A.J., Jafari R. (2011). Study of a third grade non-Newtonian fluid flow between two parallel plates using the multi-step differential transform method. Comput. Math. Appl..

[16] Rashidi M.M., Shahmohamadi H., Dinarvand S. (2008). Analytic approximate solutions for unsteady two-dimensional and axisymmetric squeezing flows between parallel plates. Math. Probl. Eng..

[17] Hoshyar H.A., Ganji D.D., Borran A.R., Falahati M. (2015). Flow behavior of unsteady incompressible Newtonian fluid flow between two parallel plates via Homotopy analysis method. Lat. Am. J. Solid. Struct..

[18] Ma B., Wang Y., Kikker A. (2019). “Analytical solutions of oscillating Couette–Poiseuille flows for the viscoelastic Oldroyd B fluid”. Acta Mech..

